# Diffusive gradients in thin films measurement of sulfur stable isotope variations in labile soil sulfate

**DOI:** 10.1007/s00216-016-9949-2

**Published:** 2016-09-29

**Authors:** Ondrej Hanousek, Jakob Santner, Sean Mason, Torsten W. Berger, Walter W. Wenzel, Thomas Prohaska

**Affiliations:** 1 Department of Chemistry - VIRIS Laboratory, University of Natural Resources and Life Sciences Vienna, Konrad-Lorenz-Strasse 24, 3430 Tulln, Austria; 2 Institute of Forest Ecology, University of Natural Resources and Life Sciences Vienna, Peter-Jordan-Strasse 82, 1190 Vienna, Austria; 3 Institute of Soil Research, University of Natural Resources and Life Sciences Vienna, Konrad-Lorenz-Strasse 24, 3430 Tulln, Austria; 4 Division of Agronomy, University of Natural Resources and Life Sciences Vienna, Konrad-Lorenz-Strasse 24, 3430 Tulln, Austria; 5 School of Agriculture, Food and Wine, University of Adelaide and the Waite Research Institute, Glen Osmond, South Australia 5064 Australia

**Keywords:** Diffusive gradients in thin films, MC ICP-MS, Sulfur, Sulfate, Isotope ratio

## Abstract

A diffusive gradient in thin films (DGT) technique, based on a strongly basic anion exchange resin (Amberlite IRA-400), was successfully tested for ^34^S/^32^S analysis in labile soil sulfate. Separation of matrix elements (Na, K, and Ca) that potentially cause non-spectral interferences in ^34^S/^32^S analysis by MC ICP-MS (multi-collector inductively coupled plasma–mass spectrometry) during sampling of sulfate was demonstrated. No isotopic fractionation caused by diffusion or elution of sulfate was observed below a resin gel disc loading of ≤79 μg S. Above this threshold, fractionation towards ^34^S was observed. The method was applied to 11 different topsoils and one mineral soil profile (0–100 cm depth) and compared with soil sulfate extraction by water. The S amount and isotopic ratio in DGT-S and water-extractable sulfate correlated significantly (*r* ^2^ = 0.89 and *r* ^2^ = 0.74 for the 11 topsoils, respectively). The systematically lower ^34^S/^32^S isotope ratios of the DGT-S were ascribed to mineralization of organic S.

## Introduction

Soluble soil sulfate is the most important sulfur (S) species in many isotopic studies, as sulfate is the dominant inorganic S form in most aerobic soils [1]. Fractionation of S stable isotopes is caused by thermodynamic and kinetic effects accompanying uptake and mineralization of S compounds by microbes and plants [1, 2], evaporation and crystallization of seawater [3], transformation of minerals [4], and other natural processes [1, 2]. The resulting variation of ^34^S/^32^S isotope ratios can be used in environmental studies to study S biogeochemistry [5], in archaeology to determine the origin of findings in burial mounds [6] or to characterize ore genesis [7].

**Fig. 1:**
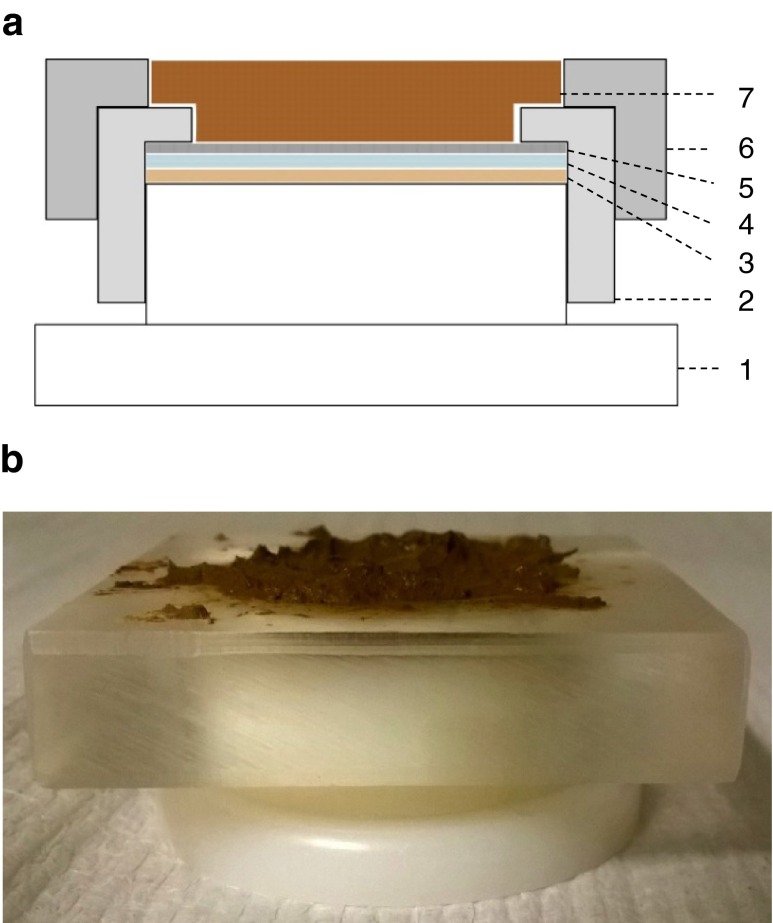
DGT sampling device schematic (**a**): *1* piston, *2* outer sleeve with sampling window, *3* resin gel, *4* diffusive gel, *5* protective membrane, *6* plastic frame to hold the soil sample in place, *7* soil sample. Application of the DGT device to soil (**b**). The figure is taken from [10] following the terms of the Creative Commons Attribution 4.0 International License (http://creativecommons.org/licenses/by/4.0/)

Multi-collector inductively coupled plasma mass spectrometry (MC ICP-MS) has been applied routinely for S isotope ratio analysis [5, 8, 9]. While high sensitivity (<0.1 μmol S required for analysis [5]) and low measurement uncertainty (<0.03 % [5]) can be achieved with MC ICP-MS, non-spectral interferences caused by matrix elements (mainly K, Na, and Ca) have been shown to be major limitations [5, 9]. Sample purification procedures have been applied successfully for overcoming matrix interferences in measurements of the sulfate-S isotopic composition in soil extracts and soil porewaters [5, 9]. Although post-sampling separation procedures are effective, they represent a time-consuming step with the potential to cause method-related isotope fractionation. A targeted sampling procedure for soil sulfate, that separates potential interferents already during the sampling step, would be an ideal alternative to conventional separation procedures. In a recent study, we developed a novel technique for passive sampling of labile soil sulfate [10], based on the diffusive gradients in thin films (DGT) methodology [8, 11]. DGT employs a solute binding agent, usually either an ion resin or a mineral binding phase (e.g., Fe oxide and Zr oxide), immobilized in a thin hydrogel layer, to sample solutes in environmental media like waters, sediments, and soils [11]. In a DGT sampler, the binding gel layer is overlain by a pure hydrogel layer and a protective membrane, which prevent particle contamination and act as diffusion layer for the solutes to be sampled (see Fig. 1).

Several studies showed the potential to use DGT for the investigation of the isotopic composition of solutes and for isotope dilution studies using radiotracers. Dalqvist et al. investigated the isotopic composition of Nd in fresh and marine waters [12], while Turner et al. analyzed ^235^U/^238^U ratios in two river waters [13]. The suitability of DGT for measuring the isotopic composition of Zn and Pb was investigated in laboratory studies [14, 15]. Sub-mm isotopic variations in dissolved sulfide-S in sediment porewaters were studied using DGT in combination with laser ablation MC ICP-MS [8, 16]. Mason et al. [17] and Six et al. [18] applied isotope dilution using the radioisotope ^32^P to compare the phosphate pool sampled by DGT and other soil test methods with the phosphate pool available for plant uptake. All of these studies concluded that DGT is well suited for measuring isotope compositions, and that the sampling process does not cause detectable isotope fractionation.

In this study, we tested and validated the measurement of the isotopic composition of labile soil sulfate-S using DGT. The method was applied to analyze the sulfate-S isotope composition of a set of mineral soil samples.

## Materials and methods

### General laboratory procedures

Laboratory tools were double acid washed using 10 % (*w*/*w*) and 1 % (*w*/*w*) HNO_3_ (p. a., Merck, Darmstadt, DE) and rinsed with laboratory water type I (0.055 μS cm^−1^; TKA-GenPure, Niederelbert, DE) before use. Laboratory water type I was also used for preparation of all standard solutions, for soil extractions, and for water saturation of soil samples. Laboratory water type I and HNO_3_ were further purified by a sub-boiling distillation system (Milestone Inc., Shelton, CT, USA) and used for the elution of sulfate from the resin gel (1 mol L^−1^ HNO_3_), from resin membranes (2 % (*w*/*w*) HNO_3_), and for microwave-assisted digestions (Multiwave 3000, Anton Paar, Graz, AT).

### DGT sampling

#### Gel and sampler preparation

DGT samplers (DGT Research Ltd., Lancaster, UK) were used for both solution and soil tests. Polyethersulfone filters (0.45 μm pore size, 0.13 mm thick, Sartorius Stedim, Goettingen, DE) were used as a protective membrane. The membranes were washed in 5 % HNO_3_ (*w*/*w*) and stored in an aqueous 10 mmol L^−1^ NaNO_3_ solution (Reagent Plus, Sigma Aldrich, Buchs, CH). Agarose cross-linked polyacrylamide (APA) diffusive hydrogels of 0.8 mm thickness were prepared according to [11] and cut to discs. Anion exchange resin (Amberlite IRA-400; chloride form, Sigma Aldrich, Buchs, CH) hydrogels for S sampling (0.4 mm thickness) were prepared according to [10]. Therefore, 3 g of the resin was ground with a ball mill for 10 min, passed through a 200-μm sieve, and washed in 10 % HCl (p.a., Merck). The resin was mixed with 10 mL gel solution [11], 60 μL of riboflavin solution (0.01 g riboflavin ((-)-Riboflavin, Sigma Aldrich) in 10 mL H_2_O), and 20 μL of tetramethylethylendiamine (TEMED; VWR Int., Randor, USA). The solution was shaken well and cast between two acid-washed glass plates (6 × 20 cm) separated by a U-shaped acid-washed plastic spacer (0.4 mm thickness). The glass plate with the freshly coated gel solution was left under fluorescent light overnight for photopolymerization. The gels were hydrated and cut to discs. A 10 mmol L^−1^ NaNO_3_ solution was used for storage of all gels.

#### Resin gel elution

After application to standard solutions or soils, the samplers were retrieved, and the resin gel was rinsed with water and eluted in 10 mL 1 mol L^−1^ HNO_3_ for 16 h. The elution efficiency (90.9 ± 1.6 %) was already reported in [10].

#### DGT performance and matrix separation

Some of the major matrix elements, like Na, K, and Ca, which are present in soil solutions at concentrations of <1 to 600 mg L^−1^ [19], may cause non-spectral interferences and, thus, affect both measurement precision and accuracy of the ^34^S/^32^S analysis by MC ICP-MS [5]. The separation of sulfate from these elements was tested using CaSO_4_ × 2 H_2_O (p.a., Fluka, Buchs, CH), K_2_SO_4_ (p.a., Fluka), and Na_2_SO_4_ × 10 H_2_O (p.a., Merck) dissolved in 3 L H_2_O to reach concentrations (*c*_Soln_) of approximately 100 mg L^−1^ S. All standard solutions had an electrolyte background concentration of 10 mmol L^−1^ NaNO_3_ (p.a., Sigma-Aldrich) and a pH value of 5.6, adjusted using dilute NaOH and HNO_3_ solutions (both p.a., Merck). DGT samplers (5 replicates) were exposed to the standard solutions for 4 h. Each experiment was repeated five times. The separation of Na, K, and Ca was calculated as difference between the cation mass fraction in standard solution (*M*_Soln_) and its mass fraction in the eluate (*M*_El_) divided by the *M*_Soln_.

To test whether uptake by DGT causes fractionation of S isotopes, two different (NH_4_)_2_SO_4_ salt batches (p.a., Merck, labelled as “A” and Normalpure, VWR, Leuven, BE, labelled as “B”) were dissolved to reach concentrations of 100 mg L^−1^ S. The two standard solutions had significantly (see Statistical analysis below) different S isotopic composition (*δ*(^34^S/^32^S)_VCDT_ “A”: 5.27 ± 0.87 ‰ (*U*; *k* = 2); *δ*(^34^S/^32^S)_VCDT_ “B”: 6.39 ± 0.64 ‰ (*U*; *k* = 2)). DGT samplers were placed into 3 L of each solution for 4 h. ^34^S/^32^S isotope ratios of the standard solutions were compared with the ^34^S/^32^S ratio of sulfate sampled by the DGT method.

In a previous study, we determined the capacity of the S resin gel to be 130 ± 11 μg S per disc (i.e., 41 ± 3 μg S cm^−2^) [10] by comparing the amount of sulfate-S bound by resin gels to the theoretical uptake onto the DGT sampler according to Eq. 1 [11]:

1 $$ {c}_{\mathrm{DGT}}=\frac{M\cdot \Delta g}{D\cdot A\cdot t} $$

where Δ*g* is the diffusive layer thickness (sum of the diffusive gel and protective membrane thicknesses), *D* is the diffusive coefficient, *A* is the sampling window surface area, and *t* is the sampling time. Elution efficiency was taken into account.

In addition to the capacity determination, we measured the isotopic composition of these DGT gel eluates to determine potential isotope fractionation in resin gels that approach analyte saturation. To account for the competition of ubiquitous anion species in soil porewaters for binding sites on the resin gel disks, a synthetic soil solution was prepared [10]. DGT samplers were deployed for 3, 6, 9, 12, 15, 24, 36, and 48 h. The ^34^S/^32^S ratio of the DGT-S was evaluated against the isotopic composition of “A” salt used for preparation of the synthetic soil solution.

Background S concentration was estimated by placing the DGT sampler into a moist plastic bag for 4 h. The mass of S eluted from this resin gel was considered a “method blank” and used for blank correction.

### Isotopic composition of labile soil sulfate

#### Soil samples

Eleven uncontaminated soils of different origin, pH, texture, and total S content (Table 1) were investigated. Jubiläumswarte and Kobernaußerwald were forest soils, Santomera originated from a research station, and the other eight samples were arable soils. In addition, samples of a forest soil profile, taken in 1996 at Brixlegg (Austria), were analyzed. This forest site had received significant amounts of S inputs from industrial SO_2_ emissions before effective flue-gas desulfurization was established. Brixlegg soil was divided into Ae, B1, B2, and two B3 horizons. For all other samples, only topsoil (max. 30 cm soil depth) samples were available. All soil samples were air dried and sieved (2 mm) before the experiment. The soil characteristics shown in Table 1 were determined according to standard procedures (pH [20], clay [21], and CaCO_3_ [22]). The water holding capacity (WHC) was determined by mixing the dried soil with water until the soil got saturated (no free water was observed). The WHC equals the mass of the water added relative to the mass of the water-saturated soil. The Brixlegg samples (BAe – BB4 samples) were evaluated separately as they represented a soil profile with the possibility to follow changes in response to the applied test methods with soil depth.

**Table 1:** Soil properties and total S content

Soil sample	Abbreviation	Country of origin	pH (CaCl_2_)	Clay g kg^−1^	WHC %	CaCO_3_ g kg^−1^	S_tot_ mg kg^−1^
Aigen	W	AT	7.1	170	43	20	200
Blankenstein	D	DE	6.2	200	39	0	252
Hohes Kreuz	H	AT	6.1	n.d.	42	0	369
Horn	R	AT	5.7	240	27	0	193
Jubiläumswarte	J	AT	5.9	180	49	0	315
Kobernaußerwald	K	AT	3.8	100	40	0	254
Moosbierbaum	B	AT	7.6	n.d.	48	100	215
Münchendorf	M	AT	7.7	n.d.	58	350	626
Santomera	E	ES	7.8	300	32	500	200
Tulln	T	AT	6.8	300	33	50	343
France	F	FR	4.8	n.d.	54	0	434
Brixlegg – Ae	BAe	AT	3.9	170	53	0	519
– B1	BB1	AT	4.0	250	42	0	199
– B2	BB2	AT	4.1	130	40	0	214
– B3a	BB3	AT	3.9	180	37	0	274
– B3b	BB4	AT	4.0	150	40	0	264

*WHC* water holding capacity

#### Total sulfur content

Of each soil sample, 0.1 g was dissolved by acid microwave-assisted digestion (5 mL sub-boiled HNO_3_ and 1 mL H_2_O_2_ (Suprapur, Merck)). The method performance was approved by digestion of the RTS-1 (CANMET, Ottawa, CA) soil reference material, certified for total and extractable S content.

#### Water extractable sulfate

Three grams of each soil sample was extracted in 18 mL laboratory water type I for 24 h (shaking over-head). The extract was filtered (Minisart RC 25, Sartorius Stedim) to remove soil particles. Anion exchange resin membranes (551642S, VWR; ionogenic group: quaternary ammonium) were applied for sulfate separation from the extract. The membranes were cleaned in 0.5 mol L^−1^ HNO_3_, and regenerated for 4 h in a 0.5 mol L^−1^ NaHCO_3_ (p.a., Merck) solution. The regenerated membranes were placed into the extract and shaken for 16 h. After rinsing the membranes with sub-boiled water, the adsorbed sulfate was extracted in 2 % HNO_3_ (18 mL) within 1 h shaking [5].

#### Sampling of DGT-labile soil sulfate-S

The soil samples were mixed with laboratory water type I to reach their maximum water holding capacity (WHC, Table 1). The resulting pastes were incubated for 24 h at 20 °C for equilibration of the soil porewater and the soil solid phase. A 2-mm-thick layer of the paste was spread carefully onto the DGT samplers, which were subsequently incubated for another 24 h at 20 °C. The eluates of the resin gels were measured for S concentration by ICP-MS and diluted to 1 mg S L^−1^ for isotopic analysis by MC ICP-MS. Uniform S concentration in samples is required for isotopic analysis to ensure a uniform ion density and exclude between-sample measurement bias. This experiment was replicated three times for each soil.

### Analyses

Single collector ICP-MS (Element XR, Thermo Fisher Scientific, Waltham, MA, USA) was used for quantification of S, Na, K, and Ca in standard solutions, resin gel eluates, and of S in soil digests and soil extracts. External calibration and internal standardization (In) were applied.

MC ICP-MS Nu Plasma HR (Nu Instruments Ltd, Wrexham, UK) connected to a sample desolvation unit (Aridus II, Teledyne, Omaha, NE, USA) was used for S isotope ratio measurement in standards and extracts. The instrument was run in edge mass resolution at (*m*/*z*)/*Δ*(*m*/*z*) ∼ 2700 (for more detail, see, e.g., [9]). The correction for the instrument background signal was performed automatically by on-peak zero measurement. External correction for instrumental isotopic fractionation (“standard-sample bracketing”) was accomplished by applying IAEA-S-1 (IAEA, Vienna, AT) as a standard. All samples and standards were diluted to 1 mg L^−1^ total S for the measurement. The gas flow rates and the lens voltage were optimized daily to reach a sensitivity of at least 5 V (mg L^−1^ sulfur)^−1^. The precision and accuracy of the measurement were assessed by measuring IAEA-S-2 (IAEA) as a sample (measurement precision: 0.2 ‰, 1 *RSD*; accuracy: long-term average of measured values: 22.53 ± 0.51 ‰ (2 SD, *n* = 22), certified value: 22.66 ± 0.20 ‰ (SD)). All values are reported as relative to a Vienna Canyon Diablo Troilite (VCDT) standard according to [23].

### Uncertainty estimation

The uncertainty estimation of the quantitative measurement (*c*_DGT_) is based on [24]. It takes the uncertainties of S quantification, diffusive layer thickness, sampling window area, sampling time, diffusion coefficient, and elution efficiency [10] as well as repeatability (SD) of the experiment into account.

The combined uncertainty of the *δ*(^34^S/^32^S)_VCDT_ measurement is based on [25]. It takes the measurement precision of the sample and of the bracketing standard, the uncertainty of the blank correction, and the correlation of ^34^S/^32^S blank signals into account. The combined uncertainty was calculated for each sample individually.

### Statistical analysis

Significance of difference between two mean values (DGT-S isotope ratios and standard solution S-isotope ratios, DGT-S isotope ratios, and water-extractable S isotope ratios) was tested with respect to the expanded uncertainties (*U*; *k* = 2) of the mean values. Two mean values were considered significantly different, if:

2 $$ \left|{m}_1-{m}_2\right| > \sqrt{U_{m1}^2+{U}_{m2}^2} $$

where *m*_1_ and *m*_2_ represent the mean values and *Um*_1_ and *Um*_2_ their expanded uncertainties [26].

Equation 3 [27] was used for computing the appropriate *t* value to test significance of a correlation coefficient (between *c*_DGT_ and water-extractable S amount and between DGT-S and water-extractable S isotope ratios) by Student’s *t* test (*p* = 0.95):

3 $$ t=r\sqrt{\frac{n-2}{1-{r}^2}} $$

where *r* is the correlation coefficient and *n* are the degrees of freedom.

## Results

### Sulfate uptake and matrix separation

Sulfate *c*_DGT_ values were in good agreement with standard solution S concentration (*c*_Soln_, see Table 2). The main contributor to the combined uncertainty of the calculated *c*_DGT_ was the repeatability of the DGT application (up to 84 %) followed by the uncertainty of the diffusion coefficient *D* (22 % on average; [10]). During sulfate sampling by DGT, the investigated matrix elements (Ca, K, and Na) remained almost entirely in the standard solution (see Table 2). The main source of the combined uncertainty was again the repeatability of the method (more than 95 %).

**Table 2:** Agreement between *c*_Soln_ and *c*_DGT_ of S and separation of cations (Na, K, and Ca) from the sulfate sampled by DGT (*n* = 5)

Standard solution	S concentration/mg L^−1^		*c*_DGT_/*c*_Soln_	Cation concentration/mg L^−1^	Cation separation
	*c*_Soln_	*c*_DGT_			(*M*_Soln_ − *M*_El_)/*M*_Soln_
Na_2_SO_4_ × 10 H_2_O	9.3 ± 0.2	8.0 ± 1.4	86 ± 15 %	14.3 ± 0.9	99 ± 1 %
K_2_SO_4_	8.9 ± 0.1	8.4 ± 0.9	95 ± 10 %	23.2 ± 1.1	99 ± 0 %
CaSO_4_ × 2 H_2_O	9.4 ± 0.2	8.9 ± 1.8	95 ± 19 %	11.5 ± 1.0	99 ± 1 %

Values with expanded uncertainties (*U*, *k* = 2)

### Sulfate-S isotope fractionation during DGT uptake

**Fig. 2:**
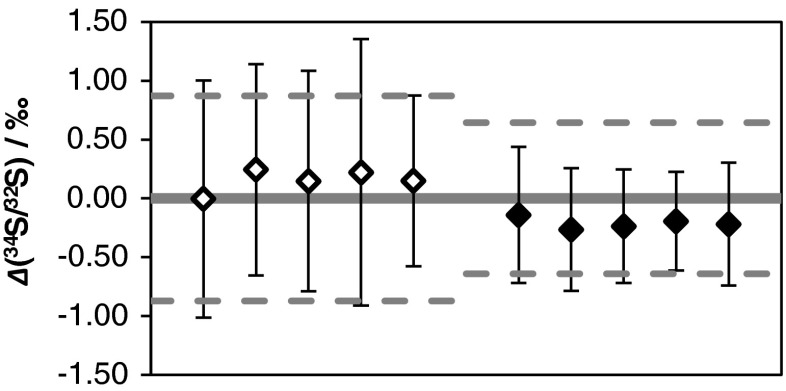
S isotope ratios measured in sulfate sampled by DGT. The values are expressed as relative to the corresponding standard solutions (batch “A” (NH_4_)_2_SO_4_: *open diamonds*, batch “B” (NH_4_)_2_SO_4_: *black diamonds*). The *error bars* and the *dashed lines* are expanded uncertainties *U*, *k* = 2

The isotopic composition of sulfate-S sampled by DGT from “A” or “B” standard solution corresponded to that of the standard solution. The typical expanded uncertainty of the *δ*(^34^S/^32^S)_VCDT_ value was ∼0.90 ‰ with measurement precision being the main contributor (up to 89 %). The different uncertainty of “A” and “B” is caused by different measurement conditions on different measurement days. The results are shown in Fig. 2. The ^34^S/^32^S isotope ratio is presented as relative to composition of the corresponding immersion solution (*Δ*(^34^S/^32^S)) for better presentation.

**Fig. 3:**
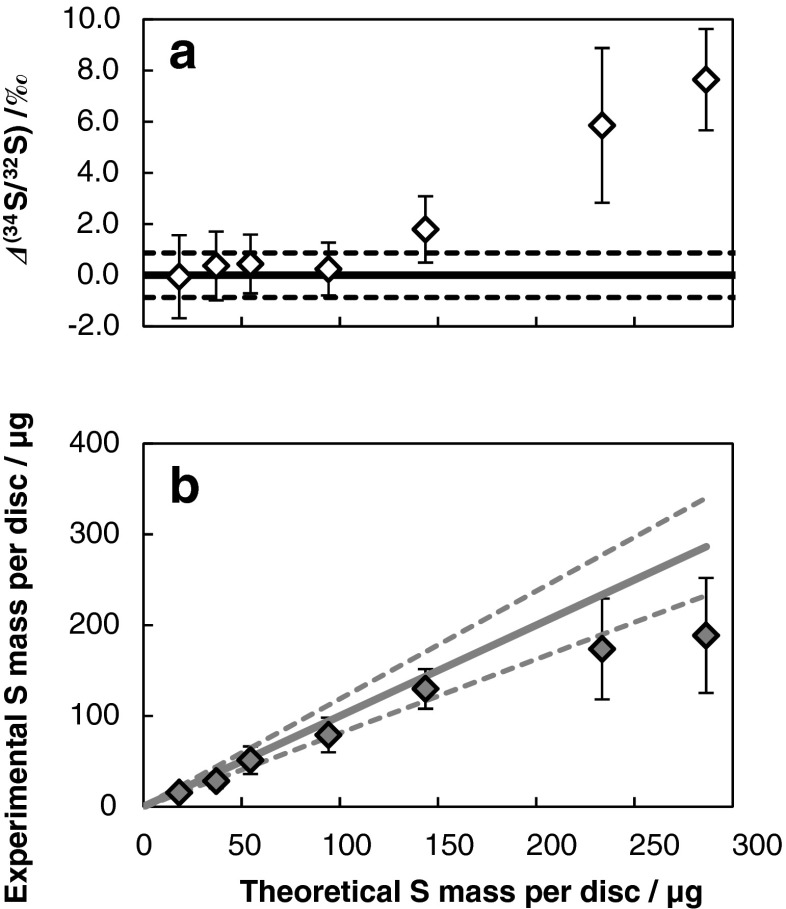
**a** *Δ*(^34^S/^32^S) isotope ratios of DTG sulfate-S (*open diamonds*) relative to synthetic soil solution (salt “A”, *black line*) with increasing resin gel loading, and **b** experimental S uptake per disc (*grey diamonds*) and theoretical (1:1) uptake line. *Error bars* and *dashed lines* are expanded uncertainties *U*, *k* = 2. More information on gel loading versus theoretical uptake can be found in Hanousek et al. [10]

The capacity experiment showed that the ^34^S/^32^S ratio of DGT-S corresponded to that of the synthetic soil solution (salt batch “A”) at gel loadings ≤ 79 μg S per disk. When the gel S loading reached and exceeded 130 μg S per disc, increased ^34^S/^32^S ratios were observed in DGT-S (Fig. 3). *Δ*(^34^S/^32^S), relative to the *δ*(^34^S/^32^S)_VCDT_ of the standard solution, was 1.79 ± 1.29, 5.85 ± 3.02, and 7.64 ± 1.98 ‰ for loadings of 130, 174, and 189 μg S per disc, respectively.

### Soil samples

**Fig. 4:**
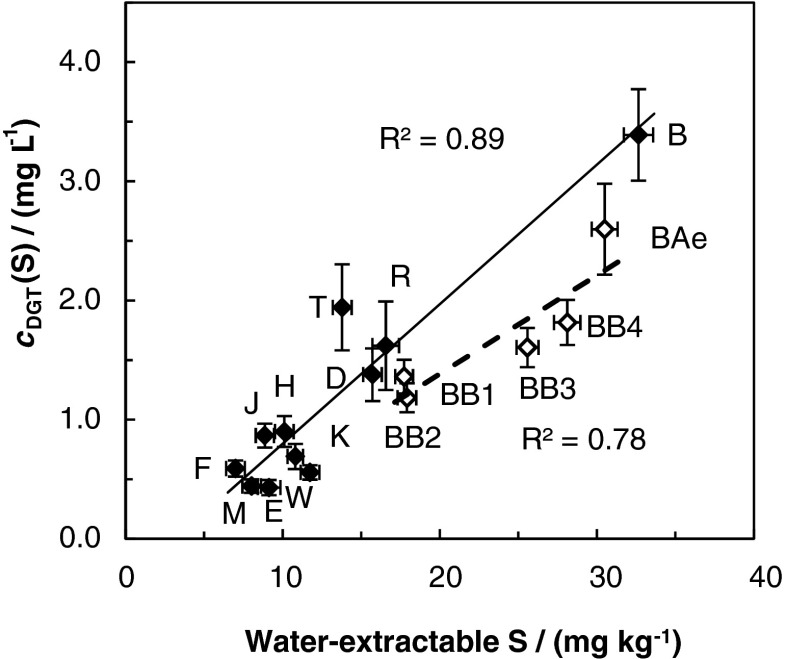
Water-extractable sulfate S concentration against the calculated S *c*_DGT_ for the 11 studied soils (*black diamonds*, *full line*) and Brixlegg soil profile (*open diamonds*, *dotted line*). The *error bars* are expanded uncertainties *U*, *k* = 2

The water-extractable S ranged between 7.0 (F) and 32.7 (B) mg S kg^−1^ soil. The minimum S *c*_DGT_ was obtained from the E sample (0.43 mg L^−1^) and the maximum from the B sample (3.39 mg L^−1^). The resin gel loadings were in the range between 9.6 μg sulfur per disc (sample E) and 76 μg sulfur per disc (sample B). The S *c*_DGT_ is plotted against the mass concentration in the soil of water-extractable S in Fig. 4. The correlation between the results was 0.89 (*r* ^2^) for the 11 studied soils and 0.78 (*r* ^2^) for the Brixlegg soil profile.

**Fig. 5:**
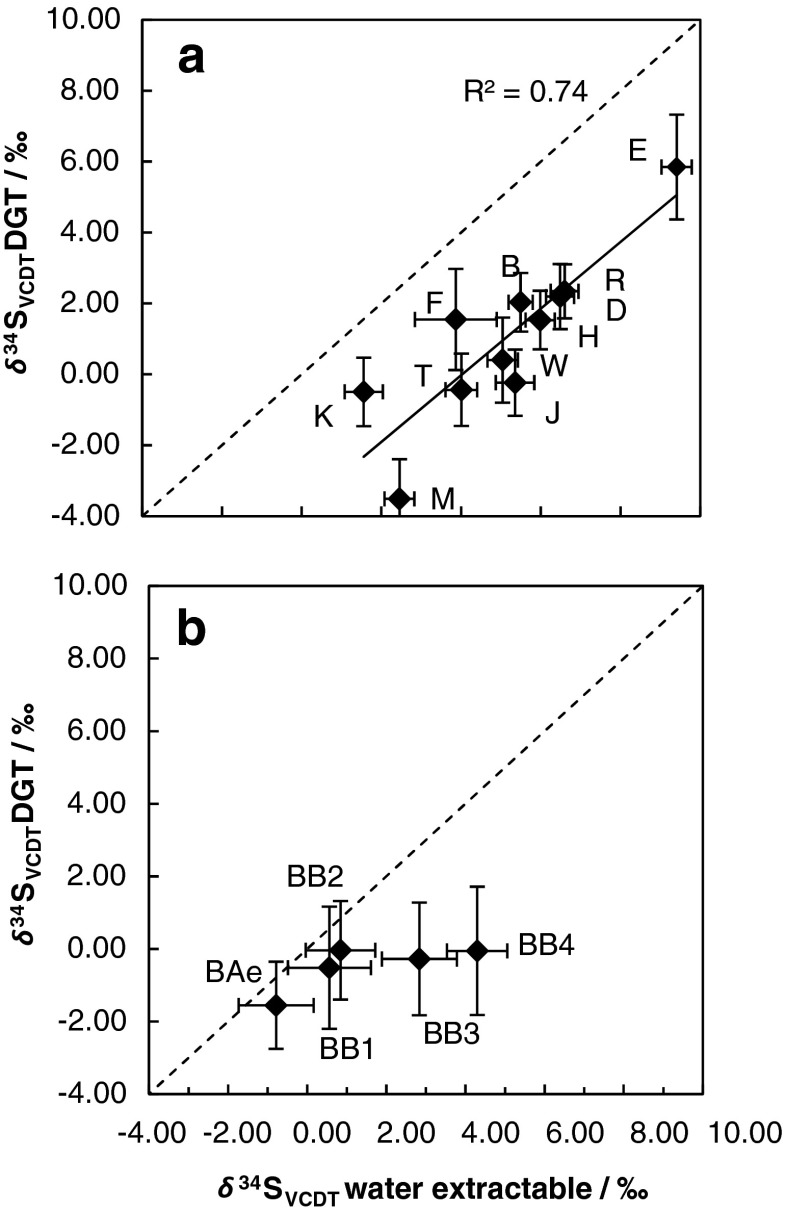
Correlation between *δ*(^34^S/^32^S)_VCDT_ values of water-extractable soil sulfate S and S sampled by DGT from **a** 11 different soils and **b** Brixlegg soil profile. *Dashed line* is the theoretical 1:1 line. *Error bars* are expanded uncertainties *U*, *k* = 2

The results of the isotopic analysis of S in water-extractable sulfate after purification by anion exchanger resin membrane and analysis of S sampled by DGT are summarized in Fig. 5. The expanded uncertainty of the *δ*(^34^S/^32^S)_VCDT_ values was 1.1 ‰ with repeatability of the experiment being the main contributor (44 %). The correlation of the two methods was 0.74 (r^2^) for the 11 soils. No correlation could be determined for the Brixlegg soil profile.

## Discussion

### Suitability of DGT for ^34^S/^32^S analysis

The non-spectroscopic matrix-based interferences in ^34^S/^32^S analysis by MC ICP-MS were discussed, e.g., by [5] or [9]. A purification step (by cation exchange column or anion exchange resin on a plastic membrane, respectively) was applied by the authors to remove matrix elements from dissolved sulfate. However, the DGT method is capable to sample dissolved sulfate selectively, thus removing the interfering matrix elements (see Table 2). While *c*_DGT_/*c*_Soln_ ratio was high for sulfate (86 ± 15 %–95 ± 19 %), all investigated matrix elements (Ca, K, and Na) were separated quantitatively (to more than 99 %) during the sampling by DGT. The main source of the relatively large expanded uncertainty *U*_cDGT_ (up to 19 %, *k* = 2) was the repeatability of the method (contributing up to 84 % to the combined uncertainty). This can be explained, e.g., by small differences (e.g., in resin amount) between resin gel batches. Thus, the DGT technique for soil S enables for a direct, matrix-free sampling of labile S, and is thus advantageous for S isotope analysis by MC ICP-MS.

The elution efficiency (90.9 ± 1.6 %) reported in [10] shows that the sulfate sampled by DGT is not completely eluted from the resin gel. Since the elution process can be accompanied by isotopic fractionation, the suitability of the DGT method for S isotopic analysis was proven.

It is evident that the comparison of the *δ*(^34^S/^32^S)_VCDT_ values in sulfate-S sampled by DGT with the values of the corresponding standard solution (“A” and “B”, Fig. 2) shows no significant isotopic fractionation. In this experiment, the mass accumulated on the gels was well below 79 μg per disc. Above this gel loading, fractionation towards ^34^S was observed (Fig. 3a). In comparison, deviation of the experimental uptake from the theoretical uptake was only observed at gel loadings >130 μg per disc (Fig. 3b [10]). Obviously, the sulfate-S isotope composition is fractionated when approaching saturation of the resin gel. Isotope fractionation on ion exchangers has been reported previously and the enrichment of ^34^S using anion exchange resin was even applied to produce compounds enriched in ^34^S [28]. Therefore, for reliably determining the sulfate-S isotope composition, the resin gel loading must not exceed 79 μg per disc, while quantitative sulfate DGT measurements with this gel are possible up to a gel loading of ≤130 μg per disc [10].

### Soil samples

The resin gel loadings in the soil deployment were generally lower than the threshold for sulfate isotope composition measurements (max. 76 μg S per disc) and thus well below the threshold for the quantitative determination of DGT-labile sulfate S (130 μg S per disc). The comparison of water-extractable sulfate S and S *c*_DGT_ showed high correlation (*r* ^2^ = 0.89) between the two techniques for the 11 different soils investigated. The correlation between the two parameters was somewhat lower (*r* ^2^ = 0.78) in the mineral soil horizons of the Brixlegg soil profile. This observation indicates that the water-extractable and DGT-labile sulfate quantities in the set of soils investigated here are closely linked. In our previous work, soil-S measured using stronger extractants (1 mol L^−1^ KCl; 1 mol L^−1^ Ca(H_2_PO_4_)_2_) yielded much lower correlations to DGT-measured S (*r* ^2^ = 0.18 and *r* ^2^ = 0.40, respectively), probably because those extractants were more efficient in extracting sorbed and mineralizable S than the H_2_O extract. Note that in the present and the earlier work [10], two different sets of soils were investigated.

The expanded uncertainty of the *δ*(^34^S/^32^S)_VCDT_ values was higher in the soil experiment compared to the experiment in laboratory solutions (0.9 and 0.5 ‰, respectively). This can be explained by small inhomogeneities of the natural samples as method repeatability was the main contributor to uncertainty of the S isotopic analysis in soils.

The results of the ^34^S/^32^S isotope ratio analysis of water-extractable sulfate S and sulfate S sampled by DGT show that DGT-S was systematically and significantly depleted in ^34^S as compared to water-extractable sulfate S. The S isotope ratios found in sulfate S sampled by the two techniques correlated significantly (*r* ^2^ = 0.74 for the 11 soils). No correlation could be determined for the Brixlegg soil profile as the *δ*(^34^S/^32^S)_VCDT_ DGT values of BAe–BB4 were not significantly different from each other (see Fig. 5b). In water-extractable sulfate *δ*(^34^S/^32^S)_VCDT_, the samples BAe–BB2 were significantly different from BB3 and BB4, while no differences were found within these two groups. A possible explanation for the water extraction revealing differences in the isotope ratios down the soil profile while DGT sampling showed no such differences could be that DGT samples a more strongly bound S fraction in soils, which is less prone to changes in the S isotopic composition than the more easily soluble S fraction sampled by the water extraction.

The general difference in the isotopic composition of the sulfur sampled by the two methods is most probably caused by the mineralization of S from organic sources during the DGT experiment, as the soil microflora prefers the lighter ^32^S isotope in metabolism [1, 29, 30]. Before DGT sampling, the soil pastes are incubated at room temperature for 24 h before and for additional 24 h during DGT sampler exposure. Therefore, a considerable amount of time is available for organic S species to be mineralized to inorganic sulfate by microbial activities. Other than in incubation/extraction schemes that are used for S mineralization studies [31], where mineralized S is extracted at the end of an incubation period, the DGT device is in close contact with moist soil and continuously binds sulfate as it gets mineralized. This feature, in combination with the possibility of varying the incubation conditions (e.g., soil moisture, temperature, incubation period, and biocidal treatment), renders DGT a potentially powerful tool for S mineralization studies, especially in comparison to conventional sampling by soil extraction.

## Conclusions

The presented DGT technique was shown to be well suitable for sulfate sampling and matrix separation in a single step, thereby avoiding the occurrence of non-spectral interferences during the MC ICP-MS measurement. Even though DGT-S can be quantified up to a resin gel disc loading of ≤130 μg S, analysis of ^34^S/^32^S is only possible up to a gel discs loading of ≤79 μg S. Such an effect has not been reported before for DGT-based methods for isotope composition measurements, but it might be important also for other resin/isotope system combinations. Therefore, we suggest that isotope fractionation vs. gel loading should be tested. Significantly and systematically lower ^34^S/^32^S isotope ratios of the DGT-S than of water-extractable sulfate-S in soils indicate mineralization of organic S during DGT application. Therefore, DGT should be a versatile tool to investigate soil S mineralization, as the experimental conditions (soil paste moisture, temperature, soil incubation/exposure time) can be modified easily. However, additional incubation tests and comparison with sulfate uptake by plants are warranted.
